# Portrait of Wm. Ferguson

**Published:** 1855-06

**Authors:** 


*Portrait of Wm. Ferguson.*—An admirer of this distinguished gentleman (so well known as the author of a system of practical surgery, and for a long time Professor of Surgery in King's College, London,) has brought to this country a few copies of an admirable lithographic portrait of him. Those wishing to procure copies of it can order them, either from Lindsay & Blakiston, Philadelphia, or from H. Bailliere, New York.

